# Geriatrics-focused indicators predict mortality more than age in older adults hospitalized with COVID-19

**DOI:** 10.1186/s12877-021-02527-w

**Published:** 2021-10-14

**Authors:** Liron Sinvani, Allison Marziliano, Alex Makhnevich, Sergey Tarima, Yan Liu, Michael Qiu, Meng Zhang, Suzanne Ardito, Maria Carney, Michael Diefenbach, Karina Davidson, Edith Burns

**Affiliations:** 1 Center for Health Innovations and Outcomes Research (CHIOR), Feinstein Institutes for Medical Research, Northwell Health, 600 Community Drive, Suite 403, Manhasset, NY 11030 USA; 2 Division of Hospital Medicine, Department of Medicine, Northwell Health, Manhasset, NY USA; 3 Donald and Barbara Zucker School of Medicine at Hofstra/Northwell, Northwell Health, Hempstead, NY USA; 4 Division of Geriatrics and Palliative Medicine, Department of Medicine, Northwell Health, Manhasset, NY USA; 5 Division of Biostatistics, Institute for Health and Equity, Medical College of Wisconsin, Milwaukee, WI USA

**Keywords:** COVID-19, Mortality, Hospital related, Outcomes, Risk factors

## Abstract

**Background:**

Age has been implicated as the main risk factor for COVID-19-related mortality. Our objective was to utilize administrative data to build an explanatory model accounting for geriatrics-focused indicators to predict mortality in hospitalized older adults with COVID-19.

**Methods:**

Retrospective cohort study of adults age 65 and older (*N* = 4783) hospitalized with COVID-19 in the greater New York metropolitan area between 3/1/20-4/20/20. Data included patient demographics and clinical presentation. Stepwise logistic regression with Akaike Information Criterion minimization was used.

**Results:**

The average age was 77.4 (SD = 8.4), 55.9% were male, 20.3% were African American, and 15.0% were Hispanic. In multivariable analysis, male sex (adjusted odds ration (adjOR) = 1.06, 95% CI:1.03-1.09); Asian race (adjOR = 1.08, CI:1.03-1.13); history of chronic kidney disease (adjOR = 1.05, CI:1.01-1.09) and interstitial lung disease (adjOR = 1.35, CI:1.28-1.42); low or normal body mass index (adjOR:1.03, CI:1.00-1.07); higher comorbidity index (adjOR = 1.01, CI:1.01-1.02); admission from a facility (adjOR = 1.14, CI:1.09-1.20); and mechanical ventilation (adjOR = 1.52, CI:1.43-1.62) were associated with mortality. While age was not an independent predictor of mortality, increasing age (centered at 65) interacted with hypertension (adjOR = 1.02, CI:0.98-1.07, reducing by a factor of 0.96 every 10 years); early Do-Not-Resuscitate (DNR, life-sustaining treatment preferences) (adjOR = 1.38, CI:1.22-1.57, reducing by a factor of 0.92 every 10 years); and severe illness on admission (at 65, adjOR = 1.47, CI:1.40-1.54, reducing by a factor of 0.96 every 10 years).

**Conclusion:**

Our findings highlight that residence prior to admission, early DNR, and acute illness severity are important predictors of mortality in hospitalized older adults with COVID-19. Readily available administrative geriatrics-focused indicators that go beyond age can be utilized when considering prognosis.

**Supplementary Information:**

The online version contains supplementary material available at 10.1186/s12877-021-02527-w.

## Background

SARS-CoV-2 infection, or Coronavirus disease 2019 (COVID-19), has impacted adults indiscriminately across the age span, yet, it has taken the greatest toll on older adults [1–5]. Of the more than 115,000,000 cases globally, 31% have been over 65 years old; accounting for 45% of hospitalizations, 53% of ICU admissions, and 80% of deaths [1, 2]. Age has consistently been implicated as one of the main risk factors for poor outcomes in COVID-19, including severe disease, hospitalization, and mortality [3–6]. While studies evaluating COVID-19 related mortality account for demographic and clinical characteristics, few have considered indicators that are highly relevant to the older adult population, namely functional and cognitive status as well as baseline advance directives regarding life-sustaining treatment preferences.

Although age in general contributes to models predicting mortality, there is robust literature demonstrating that function and frailty are strong predictors of poor outcomes among older adults [7–9]. This has also been shown in recent studies evaluating frailty in patients with COVID-19 [10, 11]. Unfortunately, functional status and frailty are infrequently incorporated into models due to insufficient assessment and documentation by medical professionals [12]. Another critical, yet frequently unaddressed and undocumented factor in older adults is advance directives, including goals of care and life-sustaining treatment preferences [13]. Early Do-Not-Resuscitate (DNR), documented within 24 h of admission, has been associated with baseline care preferences and prognosis that may not be related to the patient’s acute illness, and has been shown to be an independent risk factor for mortality [14–16]. Therefore, an early DNR can be used as a proxy for mortality risk. However, COVID-19 studies evaluating mortality have generally either excluded or failed to account for patients with DNR documentation on admission [3–5].

As COVID-19 continues to impact older adults globally, concerns about present and future shortages in hospital and critical care beds, staffing, and treatments (e.g. ventilators and vaccines), have focused attention on resource allocation. Decisions about resource allocation during COVID-19 first started in Northern Italy, where the number of sick patients grossly outnumbered available health care resources [17]. Since then, many countries and states have prepared or activated pre-existing crisis standards of care. Some of these strategies highlight older age as the main criteria, placing older adults at a disadvantage regardless of their baseline characteristics [18–25]. In a position statement regarding resource allocation during COVID-19, the American Geriatrics Society (AGS) indicated that age should never be used as the main indicator to exclude older adults from receiving care [26].

The objective of this study was to utilize readily available administrative data that incorporates geriatrics-focused indicators to build an exploratory model to predict mortality in hospitalized older adults during the initial peak of the COVID-19 pandemic.

## Methods

The study was conducted at a large academic health system serving approximately 11 million people in the greater New York metropolitan area. The COVID-19 Research Consortium and institutional review board approved the study. Data was abstracted from inpatient electronic medical records with the following inclusion criteria: age greater than or equal to 65; admitted to one of eleven health system hospitals between March 1st, 2020 and April 20th, 2020; and confirmed diagnosis of COVID-19 infection by positive result on polymerase chain reaction of a nasopharyngeal sample. Clinical course and outcomes were monitored until June 10th, 2020. Transfers from one in-system hospital to another were merged and considered as a single visit. For patients with multiple admissions during the study period, only the first admission was included. Data were collected from enterprise electronic health record (EHR) (Sunrise Clinical Manager; Allscripts). De-identified data can be provided upon request.

### Data elements

All data elements underwent a vigorous process (Sinvani, Qiu, Yan, Marziliano, Makhnevich) of data harmonization–ensuring that the data fields represent clinically relevant information. An iterative process was undertaken that consisted of a recurring loop of query, refinement, data validation by manually reviewing randomly selected charts, and inter-rater agreement, followed by further query refinement, until variable accuracy was achieved. In addition, the team hand-searched a random selection of charts to ensure data quality and integrity. Clinical members of the research team led quality control and data validation of clinical variables including comorbidity index, baseline functional status, preferences for life-sustaining treatments and discharge disposition, Modified Early Warning System (MEWS), and oxygen requirement (including mechanical ventilation).

Patient demographics included age, sex, race, ethnicity, insurance, and body mass index (BMI); all elements were captured from pre-specified categories in the EHR. BMI was categorized as underweight (< 18.5), normal (18.5 to 24.9), overweight (25 to 29.9), and obese (≥ 30). Comorbid conditions were collected using ICD-9/10 codes based on past medical history documented prior to or at index admission, including: diabetes mellitus, hypertension, chronic obstructive pulmonary disease, interstitial lung disease, asthma, cancer, coronary artery disease, atrial fibrillation, chronic kidney disease, and dementia. Comorbidity index was calculated based on the Charlson Comorbidity Index (CCI), excluding the age component [27]. Residing in a facility prior to admission was used as a surrogate indicator for functional status or the need for assistance in activities of daily living. Baseline functional status was coded as “residence prior to admission” home versus any facility (skilled nursing facility, SNF, assisted living, and group home). Further details on type of facility and level of assistance provided (e.g. long-term care vs. sub-acute rehabilitation) is not consistently documented in most EMR and was not available.

Patient preferences for life-sustaining treatments on admission were based on the presence of an “early” (within 24 h of admission) do-not-resucssitate (DNR) order. Early DNR has been described in the literature as a surrogate for worse baseline prognosis as well as preferences for life-sustaining treatment and has been shown to be an independent predictor of mortality [14–16].

Illness acuity on presentation consisted of the first documented MEWS, first documented method of oxygen delivery (no oxygen, nasal cannula, venturi mask, nonrebreather mask, high flow, noninvasive ventilation, and mechanical ventilation), and temperature on arrival (no fever vs. fever). MEWS is pre-calculated in the EHR and includes respiratory rate (breaths/min), oxygen saturation, temperature, systolic blood pressure (SBP), heart rate (HR), and level of consciousness [28]. MEWS was categorized as non-critically ill (0-4) and critically-ill (> 4) [29–31]. Certain methods of oxygen delivery (venturi masks, high flow oxygen, noninvasive mechanical ventilation) were discouraged within the health system to minimize risk of aerosolized spread and so were rarely used. They were categorized as follows: venturi mask (*n* = 30, 0.6%) was grouped with nasal cannula, and high flow oxygen (*n* = 16, 0.3%) and non-invasive ventilation (*n* = 26, 0.5%) with nonrebreather masks. Fever on arrival was defined as temperature of > 37.8 degrees Celsius.

Data elements for hospital outcomes included: 1) length of stay (LOS); 2) discharge disposition (home-home, home hospice, against medical advice vs. facility-skilled nursing facility/assisted living, psychiatric facility, hospice facility); and 3) 30-day hospital readmission.

The primary outcome of interest was hospital mortality. There were no missing values for the majority of the variables including the outcome variable. The only variables with a significant number of missing values used in the analysis were BMI (30% missing) and MEWS (9% missing).

### Analysis

**Fig. 1:**
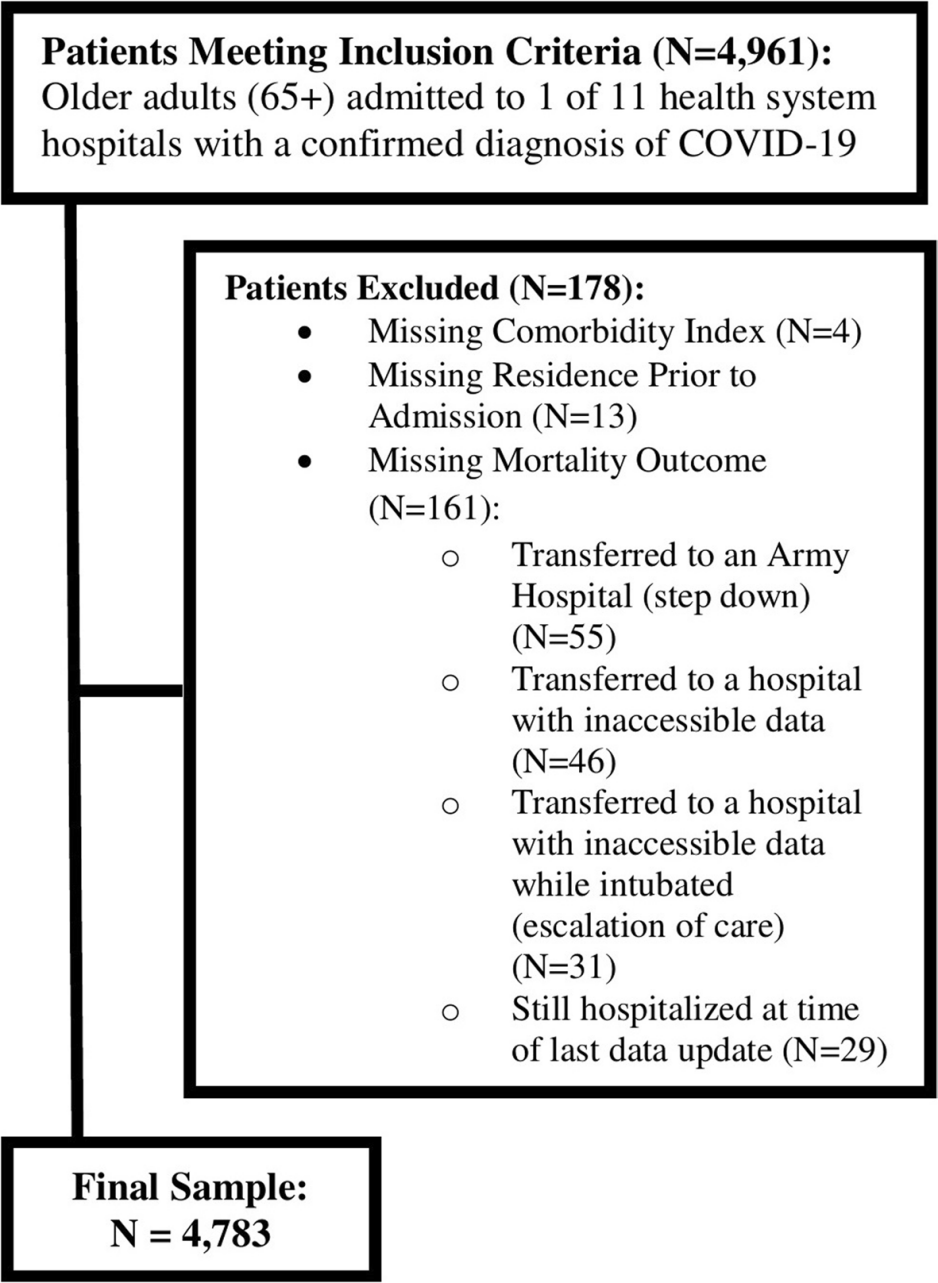
Flow Diagram of Patient Population (See Figure File)

Means and standard deviation (SD) were used to summarize distributions of continuous variables and frequencies and percentages were used for categorical variables. Patients who were still hospitalized at time of data truncation (4/20/20) or transferred to a facility in which data for their complete hospital course could not be obtained, and those with a missing comorbidity index or residence prior to admission were excluded from the unadjusted and adusted analyses (Fig. 1).

The objective was to build an explanatory model with good predictive properties that helps to explain the impact of predictors of mortality. Initial analysis was conducted with a series of simple logistic regression analyses to evaluate patient factors related to hospital mortality. These factors included: age, sex, race, ethnicity, insurance, individual comorbidities, BMI, comorbidity index (total comorbidity index excluding age contribution), residence prior to admission – home vs. facility (as a surrogate for baseline functional status), DNR order within 24 h of admission (as a surrogate for preferences for declining life-sustaining treatment), and severity of acute illness (first documented MEWS and oxygen delivery method).

Stepwise logistic regression with Akaiike Information Criterion (AIC) minimizationwas used to choose the parcimonious model. Stepwise variable selection was then applied to the entire data set of *N* = 4783 (only two variables BMI and MEWS had missing data: 1438 missing BMI; 473 missing MEWS). Two multiple imputation models minimizing AIC were found: (1) a multinomial regression model for missing BMI (4 BMI categories: underweight, normal, overweight, obese) and (2) a logistic regression model for missing MEWS (2 categories: non-critically ill and critically ill). Using multiple imputations, 50 imputed data sets were created. Then, Rubin’s approach was used to combine results from the 50 logistic regression analyses [32]. Modelling was expanded to test interactions with two linear predictors (age and comorbidity index). Multicollinearity was evaluated using variance inflation factor (VIF). Analyses were performed using SPSS v26, IBM Corp, Armonk, NY, and SAS 9.4, SAS Institute Inc., Cary, NC.; R 4.0.3 (www.p-project.com) was used to apply machine learning methods.

## Results

### Patient characteristics

A total of 4783 patients 65 years and older were included, with mean age of 77.4 years (SD = 8.4), 55.9% male; 47.4% were white, 20.3% African American, and 15.0% Hispanic. The most common comorbidities were hypertension (61.1%), diabetes (36.6%), chronic kidney disease (16.5%), atrial fibrillation (14.9%), and dementia (13.2%). Average comorbidity index (CCI without the age component) was 3.4 (± 2.9). In addition, 21.2% came to the hospital from a facility, and 5.8% had a DNR order within 24 h of admission. On arrival, the average MEWS was 3.7 (SD = 2.0) and 79.5% required oxygen therapy. See Table 1 for full details of patient characteristics.

**Table 1:** Patient Characteristics and Hospital Outcomes (*N* = 4783)

Patient Characteristics and Hospital Outcomes	M (SD) or n (%)
**Age**	77.4 (8.4) (range 65-107)
**Sex**	
Male	2673 (55.9)
Female	2110 (44.1)
**Race**	
Black	971 (20.3)
Asian	388 (8.1)
White	2267 (47.4)
Other	971 (20.3)
Not available	186 (3.9)
**Ethnicity**	
Hispanic/Latino	716 (15.0)
Not Hispanic/Not Latino	4067 (85.0)
**Insurance**	
Medicaid/Uninsured	308 (6.4)
Medicare/Private Insurance	4475 (93.6)
**Comorbidities**	
Diabetes Mellitus	1749 (36.6)
Hypertension	2924 (61.1)
Chronic Obstructive Pulmonary Disease	415 (8.7)
Interstitial Lung Disease	265 (5.5)
Asthma	239 (5.0)
Cancer	419 (8.8)
Coronary Artery Disease	261 (5.5)
Atrial Fibrillation	713 (14.9)
Chronic Kidney Disease	787 (16.5)
Dementia	629 (13.2)
**Body Mass Index**	
Underweight (below 18.5)	104 (2.2)
Normal (18.5 to 24.9)	1016 (21.1)
Overweight (25 to 29.9)	1242 (26.0)
Obese (30 or above)	983 (20.6)
Missing	1438 (30.1)
Comorbidity Index*^a^	3.4 (SD 2.9) (range 0-20)
**Residence Prior to Admission**	
Home	3769 (78.8)
Facility	1014 (21.2)
**Advance Directives**	
Early DNR^b^ (within 24 h of admission)	279 (5.8)
**Severity of Acute Illness**	
Modified Early Warning Score (MEWS)	3.7 (2.0)
Oxygen Therapy on Arrival	
None	980 (20.5)
Nasal Cannula	3133 (65.5)
Nonrebreather	453 (9.5)
Mechanical Ventilation	217 (4.5)
**Hospital Outcomes**	
Length of Stay (days)	9.9 (9.5)
Median (days)	7
Hospital Mortality	1738 (36.3)
Discharge Disposition	
Home	2011 (42.1)
Facility	1034 (21.6)
Expired	1738 (36.3)
30-Day readmission	325 (6.8)

*^a^ = excluding age component

^b^ *DNR* Do Not Resuscitate

### Hospital outcomes

The median length of stay was 7 days (mean 9.9, SD = 9.5), 36.3% expired, 42.1.6% were discharged home, and 21.6% were discharged to a facility. See Table 1 for details on hospital presentation and outcomes.

#### Univariate analysis (*N* = 4783)

Each of the following patient characteristics were related to mortality in the univariate analysis (Table 2): older age (65+, OR = 1.04 95% CI:1.03-1.05, odds of death expected to increase 4% with one- year increase in age) and male sex (OR = 1.31, 95% CI:1.17-1.48, relative to female sex) were significantly associated with outcome of death, as was admission from a facility (OR = 1.94, 95% CI:1.68-2.23, relative to living at home). In terms of medical variables, higher comorbidity index (OR = 1.08, 95% CI:1.06-1.11, odds of death expected to increase 8% with one-point increase in the score), chronic kidney disease (OR = 1.56, 95% CI:1.34-1.82), atrial fibrillation (OR = 1.53, 95% CI:1.31-1.80), dementia (OR = 1.60, 95% CI:1.35-1.89, relative to no dementia), coronary artery disease (1.63, 95% CI:1.27-2.10), cancer (OR = 1.26, 95% CI:1.03-1.54), chronic obstructive pulmonary disease (OR = 1.37, 95% CI:1.12-1.68), and interstitial lung disease (OR = 3.97, 95% CI:3.12-5.06) were all significantly associated with mortality. Markers of severity of acute illness – MEWS (OR = 6.79, 95% CI:5.90-7.80, severely-ill relative to not severely-ill), first documented oxygen delivery (OR = 2.58, 95% CI: 2.16-3.08, nasal cannula relative to room air; OR = 5.75, 95% CI: 4.49-7.36, nonrebreather relative to room air; OR = 14.67, 95% CI: 10.30-20.88, mechanical ventilation relative to room air), and life-sustaining preferences (OR = 3.50 95% CI: 2.71-4.50, having an early DNR relative to late or no DNR) were all significantly related to mortality. In contrast to previously published series, race, ethnicity, insurance status, diabetes mellitus, and BMI were not significantly related to mortality [3, 4]. Additional univariable analyses of age and the other patient characteristics can be found in Supplemental Table 1.

**Table 2:** Single Predictor Logistic Regressions of Patient Characteristics to Predict Hospital Mortality (*N* = 4783)(Expired versus Discharged Alive)

Patient Characteristics	Expired M (SD) or n (%)	Discharged Alive M (SD) or n (%)	OR (95% CI)	***P***-value
**Age**	79.20 (8.48)	76.31 (8.20)	1.04 (1.03, 1.05)^a^	< 0.001^*^
**Sex**				
Male	1045 (39.09%)	1628 (60.91%)	1.31 (1.17, 1.48)	< 0.001^*^
Female	693 (32.84%)	1417 (67.16%)	1 (ref)	
**Race**				
Black	304 (31.31%)	667 (68.69%)	0.73 (0.63, 0.86)	< 0.001^*^
Asian	164 (42.27%)	224 (57.73%)	1.18 (0.95, 1.47)	0.142
White	869 (38.33%)	1398 (61.67%)	1 (ref)	–
Other	336 (34.60%)	635 (65.40%)	0.85 (0.73, 0.99)	0.04^*^
Not available	65 (34.95%)	121 (65.05%)	0.86 (0.63, 1.18)	0.361
**Ethnicity**				
Hispanic or Latino	242 (33.80%)	474 (66.20%)	0.87 (0.74, 1.03)	0.12
Not Hispanic or Latino	1403 (36.87%)	2402 (63.13%)	1 (ref)	
**Insurance**				
Medicaid or Uninsured	108 (35.06%)	200 (64.94%)	0.94 (0.74, 1.20)	0.63
Medicare or Private	1630 (36.35%)	2854 (63.65%)	1 (ref)	
**Comorbidities**				
Diabetes Mellitus				
Yes	662 (37.85%)	1087 (62.15%)	1.11 (0.98, 1.25)	0.10
No	1076 (35.46%)	1958 (64.54%)	1 (ref)	
Hypertension				
Yes	1008 (58.00%)	730 (42.00%)	0.814 (0.72, 0.92)	< 0.001^*^
No	1916 (62.92%)	1129 (37.08%)	1 (ref)	
Chronic Obstructive Pulmonary Disease				
Yes	179 (43.13%)	236 (56.87%)	1.37 (1.12, 1.68)	0.003^*^
No	1559 (35.69%)	2809 (64.31%)	1 (ref)	
Intertitial Lung Disease				
Yes	216 (67.29%)	105 (32.71%)	3.97 (3.12, 5.06)	< 0.001^*^
No	1522 (34.11%)	2940 (65.89%)	1 (ref)	
Asthma				
Yes	81 (33.89%)	158 (66.11%)	0.89 (0.68, 1.18)	0.420
No	1657 (36.47%)	2887 (63.53%)	1 (ref)	
Cancer				
Yes	176 (41.31%)	250 (58.69%)	1.26 (1.03, 1.54)	.03^*^
No	1562 (35.85%)	2795 (64.15%)	1 (ref)	
Coronary Artery Disease				
Yes	124 (47.51%)	137 (52.49%)	1.63 (1.27, 2.10)	< 0.001^*^
No	1614 (35.69%)	2908 (64.31%)	1 (ref)	
Atrial Fibrillation				
Yes	321 (45.02%)	392 (54.98%)	1.53 (1.31, 1.80)	< 0.001^*^
No	1417 (34.82%)	2653 (65.18%)	1 (ref)	
Chronic Kidney Disease				
Yes	356 (45.24%)	431 (54.76%)	1.56 (1.34, 1.82)	< 0.001^*^
No	1382 (34.58%)	2614 (65.42%)	1 (ref)	
Dementia				
Yes	290 (46.10%)	339 (53.90%)	1.60 (1.35, 1.89)	< 0.001^*^
No	1448 (34.86%)	2706 (65.14%)	1 (ref)	
**BMI**				
Underweight BMI below 18.5	46 (44.23%)	58 (55.77%)	1.44 (0.96, 2.16)	0.080
Normal 18.5 to 24.9	361 (35.53%)	655 (64.47%)	1 (ref)	
Overweight, 25 to 29.9	421 (33.90%)	821 (66.10%)	0.93 (0.78, 1.11)	0.417
Obese, 30 or above	323 (32.86%)	660 (67.14%)	0.89 (0.74, 1.07)	0.208
**Comorbidity Index** (CCI-age, continuous)^b^	3.85 (2.97)	3.18 (2.76)	1.08 (1.06, 1.11)	< 0.001^*^
**Residence Prior to Admission**				
Facility	495 (48.82%)	519 (51.18%)	1.94 (1.68, 2.23)	< 0.001^*^
Home	1243 (32.98%)	2526 (67.02%)	1 (ref)	
**Advanced Directives**				
Early DNR^c^				
Yes	181 (64.87%)	98 (35.13%)	3.50 (2.71, 4.50)	< 0.001^*^
No	1557 (34.57%)	2947 (65.43%)	1 (ref)	
**Severity of Illness on Presentation**				
MEWS ^d^				
Severely-ill	993 (68.15%)	464 (31.85%)	6.79 (5.90, 7.80)	< 0.001^*^
Not severely-ill	684 (23.97%)	2169 (76.03%)	1 (ref)	
**First documented oxygen**				
Nasal cannula^e^	1140 (36.39%)	1993 (63.61%)	2.58 (2.16, 3.08)	< 0.001^*^
Nonrebreather^f^	254 (56.07%)	199 (43.93%)	5.75 (4.49, 7.36)	< 0.001^*^
Mechanical ventilation	166 (76.50%)	51 (23.50%)	14.67 (10.30, 20.88)	< 0.001^*^
Room air	178 (18.16%)	802 (81.84%)	1 (ref)	–

^a^ Odds of death expected to increase 4% with one- year increase in age

^b^ *CCI* Charlson Comorbidity Index

^c^ *DNR* Do Not Resuscitate

^d^ *MEWS* Modified Early Warning Score

^e^ Nasal cannula includes ventimask

^f^ Nonrebreather includes high flow and non-invasive ventilation

^*^ *p* ≤ 0.05

#### Multivariable analyses (*N* = 4783)

In the multivariable analysis (Table 3), we found that main effects of male sex (adjusted odds ration, adjOR:1.06, 95% CI:1.03-1.09, *p* < 0.001), Asian race (adjOR = 1.08, 95% CI:1.03-1.13, *p* < 0.001); low/normal BMI (adjOR:1.03, 95% CI:1.00-1.07, *p* = 0.03, relative to overweight/obese); higher comorbidity index (adjOR = 1.01, 95% CI:1.01-1.02, *p* < 0.001); admission from a facility (adjOR = 1.14, 95% CI:1.09-1.20, *p* < 0.001, relative to admission from home); past medical history of chronic kidney disease (adjOR = 1.05, 95% CI:1.01-1.09, *p* = 0.01), and interstitial lung disease (adjOR = 1.35, 95% CI:1.28-1.42, *p* < 0.001); early Do-Not-Resuscitate (DNR, declining life-sustaining treatment preferences, adjOR = 1.38, 95% CI:1.22-1.57, *p* < 0.001, relative to late or no DNR); higher illness severity including higher MEWS (adjOR = 1.47, 95% CI:1.40-1.54, *p* < 0.001, relative to not severely-ill; and higher oxygen requirements (adjOR = 1.17, 95% CI:1.14-1.21, *p* < 0.001, nasal cannula relative to room air; adjOR = 1.34, 95% CI:1.28-1.40, *p* < 0.001, nonrebreather relative to room air adjOR = 1.52, 95% CI:1.43-1.62, *p* < 0.001, mechanical ventilation relative to room air), were associated with mortality. After variable selection driven by AIC minimization, main effects of age, dementia, and some individual comorbid conditions (e.g., diabetes) were found not to be independently associated with mortality after controlling for the other variables, and therefore not included in the final model. While the main effect of age was not an independent predictor of mortality, it had a positive interaction (increased mortality with age) with MEWS (non-critically ill, adjOR = 1.04, 95% CI:1.01-1.07, *p* = 0.012), and negative interaction (decreased mortality with age) with a history of hypertension (adjOR = 0.98, 95% CI:0.94-0.99, *p* = 0.015) and early DNR (adjOR = 0.93, 95% CI:0.87-0.98, *p* = 0.012). For example, the main effect of hypertension is described by adjOR = 1.02, however, there was a negative interaction with age, described by adjOR = 0.97, such that for a 65-year-old, the adjOR of hypertension was 1.02, but for a 75-year-old, the adjOR = 0.99; and for an 85-year-old, the adjOR = 0.96. In the above multiple logistic regression analysis, age was centered at 65 years and rescaled to a unit change of 10 years.

**Table 3:** Patient Factors Significantly Associated with Hospital Mortality (Multiple Logistic Regression, C-Statistics = 80.6%)

Patient Characteristics	Adjusted OR (95% CI)	***p***-value
Male	1.06 (1.03, 1.09)	< 0.001
Asian	1.08 (1.03, 1.13)	< 0.001
Hypertension	1.02 (0.98, 1.07)	0.268
Chronic Kidney Disease	1.05 (1.01, 1.09)	0.008
Interstitial Lung Disease	1.35 (1.28, 1.42)	< 0.001
BMI < 25	1.03 (1.00, 1.07)	0.030
Comorbidity Index^a^	1.01 (1.01, 1.02)	< 0.001
Admission from facility	1.14 (1.09, 1.20)	< 0.001
Early DNR^b^	1.38 (1.22, 1.57)	< 0.001
MEWS^c^ > 4 (severely-ill)	1.47 (1.40, 1.54)	< 0.001
Nasal Cannula^d^	1.17 (1.14, 1.21)	< 0.001
Nonrebreather^d^	1.34 (1.28, 1.40)	< 0.001
Mechanical Ventilation^d^	1.52 (1.43, 1.62)	< 0.001
Effect of Age if history of Hypertension^e^	0.97 (0.94, 0.99)	0.015
Effect of Age if early DNR^e^	0.92 (0.87, 0.98)	0.007
Effect of Age if Admission from a Facility^e^	0.97 (0.95, 1.00)	0.063
Effect of Age if MEWS ≥4 (Critically-Ill)^e^	0.96 (0.94, 0.99)	0.012

^a^ Comorbidity Index = Charlson Comorbidity Index (without age component)

^b^ *DNR* Do-Not-Resuscitate

^c^ *MEWS* Modified Early Warning Score

^d^ First Documented Oxygen Delivery

^e^Age is centered at 65 years of age and rescaled to a unit of 10 years

C-statistic for the final model was 80.6%, which indicated this was a good model to predict mortality. VIFs for all the variables in the model were around 1, so that multicollinearity was not a cause of concern for this model. (Supplemental Table 2).

### Sensitivity analyses

In logistic regression analysis, the direction and significance of the association with mortality did not change when missing data on BMI and MEWS were treated as missing data categories.

## Discussion

Our study found that in hospitalized older adults with COVID-19 infection, male sex, Asian race, admission from a facility, presence of an early DNR, higher multimorbidity and illness severity were predictive of increased mortality. Our study found that sex, multimorbidity, residence prior to admission, early DNR, and illness severity were predictive of hospital mortality. While age was not an independent predictor of mortality, we observed several interactions of age with factors predicting mortality: increasing age was negatively associated with mortality in patients with hypertension and early DNR, and age was positively associated with mortality for patients residing at home prior to admission and those that were not critically-ill. In other words, the impact of age on mortality is minimal in the setting of severely critical disease and other factors indicating highly impaired function. These findings highlight the importance of considering readily available geriatrics-focused indicators, other than age, when considering prognostication and management, especially in the context of a pandemic when medical resources are in danger of being overwhelmed. Therefore, finding predictors other than age would be beneficial in preventing ageism.

There is robust literature demonstrating that baseline functional status is strongly associated with poor outcomes among older adults [7, 8]. However, baseline functional status is infrequently assessed at hospital admission. Medical professionals often rely on subjective impressions of function, which have been shown to be inaccurate [12]. Furthermore, even when assessed, functional status is rarely documented in a meaningful or consistent fashion [12]. While there is increasing evidence demonstrating an association between frailty and poor outcomes in those with COVID-19 [10, 11, 33], these measures may not be readily available on hospital presentation nor captured in a systematic fashion in most EHRs (especially if no previous admissions exist) [34], as is the case in our health system. During the first surge of the COVID-19 crisis, health systems may utilize shortened, “emergency documentation” protocols, which do not include functional measures such as activities of daily living. Truncated protocols are often used in emergency situations when large volumes of patients are admitted within a short period of time. Administrative data based frailty scores may also be used, but have shown only fair to moderate agreement with clinical frailty scales [35].

Our study utilized the ‘residence prior to admission’ variable as a surrogate marker of the need for skilled care- baseline functional status (arriving from a facility prior to admission). Although we were unable to distinguish between those who were “life-stay” residents in long-term care versus limited-stay for sub-acute rehabilitation, all of these locations house populations who generally have functional impairments and/or lack adequate support in the home setting. Our findings differ from a previous study from Spain, which demonstrated that retirement facilities were not associated with mortality during the first wave in adjusted models. However, the total number of COVID cases reported in this series was small, 239, just over 60% were over age 60 (~ 145), and only 26 resided in a retirement facility, suggesting this cohort would be underpowered to detect such differences. While it is important for future studies to evaluate baseline functional status using more precise, objective measures, this is often difficult in the real-world setting. Therefore with further validation, ‘residence prior to admission’ may be a good surrogate marker that can be easily obtained. Assessing and incorporating functional status is essential when evaluating clinical outcomes in older adults.

Another critical factor that must be accounted for is advance directives, including discussion and documentation of life-sustaining treatment preferences and DNR orders. Provider predictions about life expectancy typically do not match the patient’s treatment preferences [13, 36]. Although age has traditionally been used as a “proxy” for life expectancy, older adults of the same age can have very different trajectories. While advance directives should be discussed openly, this discussion and decision should not be imposed or pressured, which is most likely to happen in an acute, emergent setting. As such, timely, patient-centered goals of care discussions that focus on what matters most to the patient are essential [37]. A recent study by Alhatem et al., demonstrated that DNR status prior to admission determines mortality in patients with COVID-19 [38]. There was a negative interaction between age and early DNR, in that early DNR was less predictive of mortality in older age. This suggests that early DNR in younger age indicates overall poor health, whereas, early DNR in older age indicates wishes that may be independent of overall health.

In response to treatment decisions and resource allocation during COVID-19, several strategies, guidelines, and frameworks have been proposed, most of which highlight age as the main factor in making resource allocation determinations [18–25, 39]. While a few of these guidelines recommend advanced age as a sole exclusion, the majority include age along with other criteria such as comorbid conditions, functional status, advance directives (specifically preferences for life-sustaining interventions), and severity of acute illness [21–24, 39]. However, due to the limited ability to account for functional status and advance directives, age often becomes the de facto factor used for resource allocation [40]. Our findings support the AGS position statement indicating age alone should never be used to make decisions regarding resource allocation under conditions of resource scarcity [26]. Although age is still an important factor in the overall risk of COVID-19 mortality through the risk of having increased baseline vulnerability and severe symptoms that require one to be hospitalized in the first place, a comprehensive approach that accounts for the above factors is essential in preventing ageism. Consideration of geriatrics-focused indicators will also be essential for future studies evaluating post-COVID sequelae, including post-discharge outcomes within and beyond 30 days [41].

This study has several limitations. First, due to the overwhelming number of cases admitted at the peak of COVID-19, our large integrated health system functioned as “one hospital.” Patients were transferred within the health system as well as to nationally-operated army facilities. While transferred patients were included in the descriptive data, they were excluded from the univariate and multivariate analyses if their discharge disposition was unknown. In addition, patients who were still admitted at the time of the last data update were also excluded. While the number of transferred and admitted patients (*N* = 161 and *N* = 29, respectively) was small and unlikely to make a difference statistically, there is still a small risk of bias. A second limitation is that given the retrospective nature of the study and large numbers of patients, we were limited to elements in the EHR and thus used surrogate markers for functional status and life-sustaining treatment preferences as well as the MEWS, which has not been evaluated in COVID-19. The advantage of utilizing administrative hospital data is that it does not rely on manual scores that must be entered by clinicians into the medical record or manual data extraction. We have previously published the validity of these data elements as surrogate markers [15].

## Conclusion

COVID-19 has disproportionally affected older adults. This study confirms findings reported by other investigators that male sex, higher comorbidity index and higher illness severity are associated with hospital mortality. The confirmation of early DNR and ‘residence prior to admission’ are important additions to the literature and should be considered in future studies when considering patient-centered outcomes research. Lastly, while age remains an important factor in the overall risk of COVID-19 mortality, through the greater likelihood of physiological vulnerability and severe symptoms leading to hospitalization, a comprehensive approach that accounts for geriatrics-focused indicators is essential when evaluating prognosis and treatment decisions for hospitalized older patients with COVID-19.

## Supplementary Information

**Additional file 1: Supplemental Table 1.** Univariate Logistic Regressions of the Association between Age and Patient Characteristics (*N* = 4783) (Expired versus Discharged Alive).

**Additional file 2: Supplemental Table 2.** VIFs for Factors Indicating No Multicollinearity in the Multivariable Logistic Regression Model for Hospital Mortality.

## Data Availability

The datasets used and/or analysed during the current study are available from the corresponding author on reasonable request.

## Abbreviations

DNR: Do-Not-Resuscitate
COVID-19: Coronavirus disease 2019
ICU: Intensive Care Unit
AGS: The American Geriatrics Society
EHR: Electronic Health Record
MEWS: Modified Early Warning Score
BMI: Body Mass Index
CCI: Charlson Comorbidity Index
SNF: Skilled Nursing Facility
LOS: Length of Stay
SD: Standard Deviation
AIC: Akeike Information Criterion
BART: Bayesian Additive Regression Trees
VIF: Variance Inflation Factor
