# Supplementary material for: Geriatrics-focused indicators predict mortality more than age in older adults hospitalized with COVID-19
Source: BMC Geriatr. 2021 Oct 14;21:554. doi: 10.1186/s12877-021-02527-w (PMC8515323; doi:10.1186/s12877-021-02527-w)
Supplement: Supplementary file 1 — Additional file 1: Supplemental Table 1. Univariate Logistic Regressions of the Association between Age and Patient Characteristics (N = 4783) (Expired versus Discharged Alive). [file 12877_2021_2527_MOESM1_ESM.docx]

**Supplemental Table 1:** Univariate Logistic Regressions of the Association between Age and Patient Characteristics (N = 4,783) (Expired versus Discharged Alive)

| Patient Factors | Age | | p-value |
| --- | --- | --- | --- |
|  | Mean | Standard Deviation (SD) |  |
| **Sex** |  |  | <0.001 |
| Male | 76.24 | 7.91 |  |
| Female | 78.84 | 8.84 |  |
| **Race** |  |  | <0.001 |
| Black | 75.87 | 7.77 |  |
| Asian | 75.70 | 7.73 |  |
| Other | 75.91 | 7.92 |  |
| Not available | 77.03 | 8.12 |  |
| White | 79.46 | 8.92 |  |
| **Ethnicity** |  |  | <0.001 |
| Hispanic or Latino | 75.78 | 7.74 |  |
| Not Hispanic or Latino | 77.78 | 8.53 |  |
| **Insurance** |  |  | <0.001 |
| Medicaid or Uninsured | 74.83 | 7.73 |  |
| Medicare or Private | 77.56 | 8.45 |  |
| **Comorbidities** |  |  |  |
| Hypertension |  |  | 0.148 |
| Yes | 77.25 | 8.29 |  |
| No | 77.61 | 8.66 |  |
| Interstitial Lung Disease |  |  |  |
| Yes | 75.37 | 6.87 | <0.001 |
| No | 77.48 | 8.49 |  |
| Diabetes Mellitus |  |  | <0.001 |
| Yes | 75.92 | 7.68 |  |
| No | 78.23 | 8.73 |  |
| Asthma |  |  | 0.004 |
| Yes | 75.86 | 7.82 |  |
| No | 77.47 | 8.46 |  |
| Chronic kidney Disease |  |  | <0.001 |
| Yes | 78.61 | 8.49 |  |
| No | 77.15 | 8.40 |  |
| Chronic Obstructive Pulmonary Disease |  |  | 0.275 |
| Yes | 77.82 | 7.96 |  |
| No | 77.35 | 8.48 |  |
| Dementia |  |  | <0.001 |
| Yes | 84.01 | 7.84 |  |
| No | 76.38 | 8.06 |  |
| **Body Mass Index (BMI)** |  |  | <0.001 |
| Underweight BMI below 18.5 | 83.18 | 9.45 |  |
| Normal 18.5 to 24.9 | 79.39 | 8.70 |  |
| Overweight, 25 to 29.9 | 76.29 | 7.84 |  |
| Obese, 30 or above | 74.21 | 6.88 |  |
| **Residence Prior to Admission** |  |  | <0.001 |
| Facility | 81.03 | 8.81 |  |
| Home | 76.39 | 8.05 |  |
| **Early Do Not Resuscitate** |  |  | <0.001 |
| Yes | 85.00 | 8.28 |  |
| No | 76.91 | 8.21 |  |
| **Severity of Illness on Presentation** |  |  |  |
| **Modified Early Warning Score** |  |  | <0.001 |
| Severely-ill (>4) | 81.39 | 8.57 |  |
| Not severely-ill (< or =4) | 75.37 | 7.72 |  |
| **First documented oxygen** |  |  | 0.007 |
| Room air | 77.61 | 8.49 |  |
| Nasal cannula | 77.36 | 8.51 |  |
| Nonrebreather | 77.95 | 8.32 |  |
| Mechanical ventilation | 75.63 | 7.10 |  |
