# Supplementary material for: Geriatrics-focused indicators predict mortality more than age in older adults hospitalized with COVID-19
Source: BMC Geriatr. 2021 Oct 14;21:554. doi: 10.1186/s12877-021-02527-w (PMC8515323; doi:10.1186/s12877-021-02527-w)
Supplement: Supplementary file 2 — Additional file 2: Supplemental Table 2. VIFs for Factors Indicating No Multicollinearity in the Multivariable Logistic Regression Model for Hospital Mortality. [file 12877_2021_2527_MOESM2_ESM.docx]

**Supplemental Table 2.** VIFs for Factors Indicating No Multicollinearity in the Multivariable Logistic Regression Model for Hospital Mortality

| **Patient Characteristics** | **VIF^d^** |
| --- | --- |
| Gender | 1.01 |
| Comorbidity Index^a^ | 1.04 |
| Residence Prior to Admission | 1.07 |
| MEWS^b^ | 1.01 |
| First documented oxygen | 1.01 |
| Early DNR^c^ | 1.03 |

^a^ Comorbidity Index = Charlson Comorbidity Index - age component

^b^ MEWS = Modified Early Warning Score

^c^ DNR = Do-Not-Resuscitate

^d^ VIF: A variance inflation factor detects multicollinearity in regression analysis. It estimates how much the variance of a regression coefficient is inflated due to multicollinearity in the model. In general, a VIF above 10 indicates high correlation and is cause for concern.
